# Use of an Amorphous Silica-Based Fiber Wound Matrix and Cream in a Post-Mohs Defect of the Dorsal Hand: A Case Report

**DOI:** 10.7759/cureus.115767

**Published:** 2026-09-04

**Authors:** Adam M Jorgensen, William L Hickerson

**Affiliations:** 1 Research and Development, SiOxMed, Winston-Salem, USA

**Keywords:** amorphous silica, dorsal hand, epithelialization, exposed tendon, granulation tissue, keratoacanthoma, mohs micrographic surgery, secondary intention healing, wound closure, wound matrix

## Abstract

Postoperative defects of the dorsal hand following Mohs micrographic surgery may present wound-management challenges because of limited soft-tissue coverage, underlying tendon exposure, mobility, and the potential for functional impairment. We report the clinical course of a 53-year-old patient who underwent Mohs surgery for a biopsy-confirmed keratoacanthoma of the dorsal hand. Following complete removal of the lesion, the surgical defect was managed by secondary intention. Initial postoperative management consisted of Puracol Plus and petrolatum, with granulation coverage of an exposed tendon anticipated within approximately seven weeks. The patient subsequently received an amorphous silica-based fiber (SBF) wound matrix and silica-based cream (SBC) from SiOxMed for home-based self-treatment. SBF was applied daily to the open wound following daily rinsing and reapplication until the wound surface was covered and dry. Serial patient-generated photographs demonstrated granulation tissue covering the previously exposed tendon by Day 26, substantial wound contraction and developing epithelial coverage by Day 45, and complete visible wound coverage by Day 50. SBC was initiated after epithelial coverage was established. A final photograph obtained on Day 118 demonstrated sustained wound closure. This single uncontrolled case cannot establish that SBF or SBC accelerated healing compared with conventional secondary-intention management. However, the observed progression from a postoperative defect with an exposed tendon to granulation coverage, contraction, epithelialization, and sustained closure supports prospective evaluation of this treatment approach in complex postoperative wounds.

## Introduction

Mohs micrographic surgery provides margin-controlled excision of cutaneous malignancies while preserving uninvolved tissue [1]. Surgical defects may be reconstructed primarily, with local flaps or grafts, or allowed to heal by secondary intention depending on the wound location, depth, patient characteristics, and reconstructive goals.

Secondary-intention healing relies on granulation tissue formation, wound contraction, and epithelialization [2]. Defects of the dorsal hand may be particularly challenging because of relatively limited soft-tissue coverage and the proximity of extensor tendons and other underlying structures [3]. When a tendon is exposed, development of a vascularized granulation bed is an important component of subsequent wound closure and may influence reconstructive options [4].

An amorphous silica-based fiber (SBF) wound matrix is a conformable, nonwoven, absorbent matrix composed of synthetic SBFs. Unlike gauze, which primarily covers the wound, or ointments, which form a topical layer, an SBF is applied directly to the wound bed, where its porous fibrous structure conforms to the defect, absorbs fluid, and provides a scaffold-like wound interface. Preclinical studies suggest that silica-based fibrous materials may support cellular migration, vascularization, and extracellular matrix remodeling [5]. However, clinical evidence describing their use in postoperative dorsal-hand defects with exposed tendons is limited. This case provides serial photographic documentation of tendon coverage, wound contraction, epithelialization, and sustained closure during sequential use of SBF and a silica-based cream (SBC). The aim of this report is to describe the clinical course, compare the observed timeline with published outcomes for secondary-intention healing of dorsal-hand defects, and identify outcomes for future prospective evaluation.

## Case presentation

A 53-year-old patient presented with a lesion on the dorsal hand that was diagnosed as a keratoacanthoma. At the time of surgery, the patient had no history of diabetes, peripheral circulatory disease, autoimmune disease, or other known condition affecting wound healing. His body mass index was approximately 29 kg/m², and he reported smoking approximately one pack of cigarettes per day. His only routine medication was pantoprazole, and he reported no use of corticosteroids, immunosuppressive medications, antiplatelet agents, or anticoagulants.

The patient underwent Mohs micrographic surgery, with histopathologic examination confirming complete removal of the lesion. The resulting postoperative defect was located on the dorsal hand, measured approximately 25mm in diameter, and involved exposure of the underlying tendon (Figure 1A).

**Figure 1:**
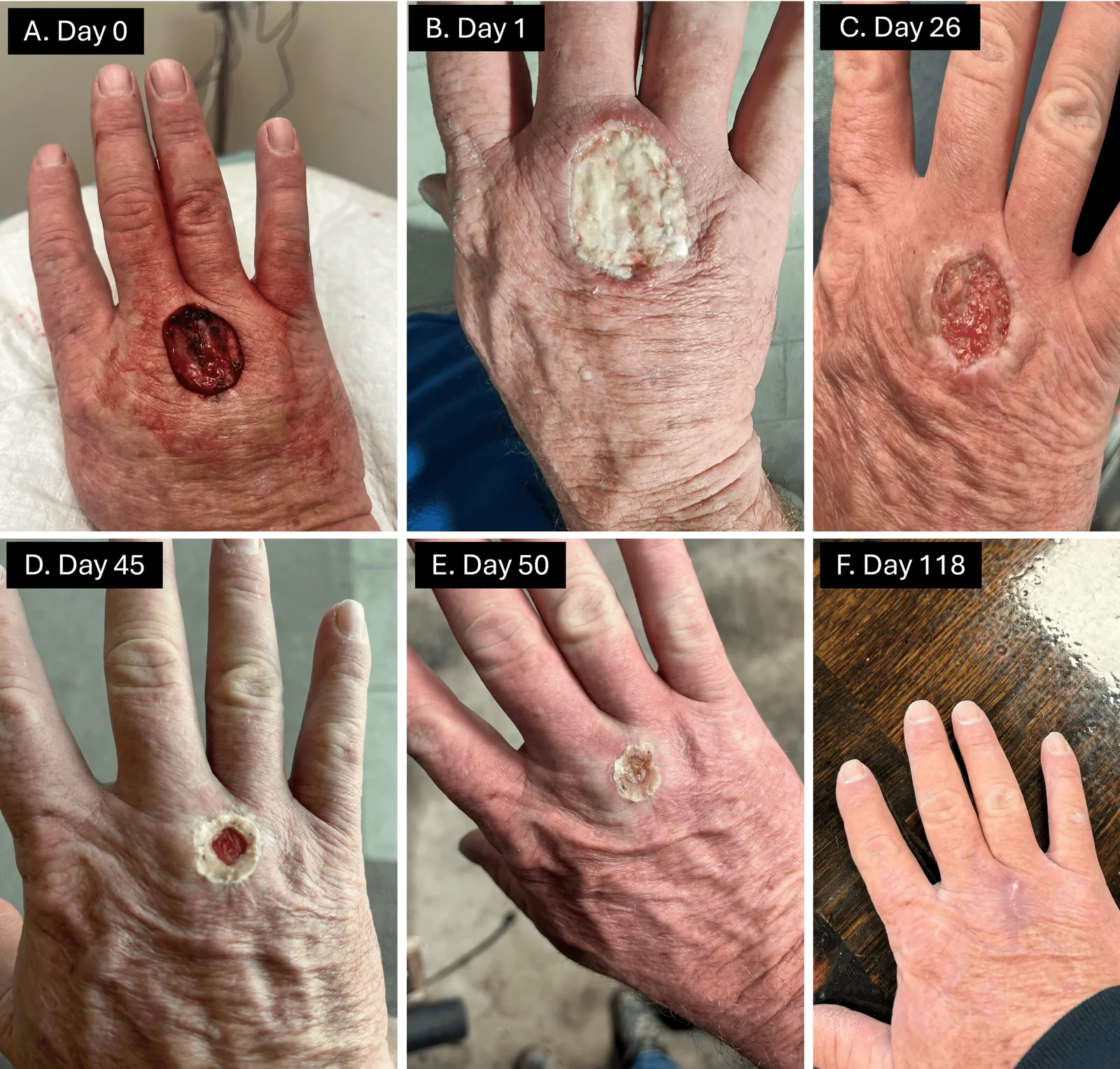
Serial appearance of a dorsal hand wound following Mohs micrographic surgery and home-based self-treatment with an amorphous silica-based fiber wound matrix and silica-based cream. A. Day 0, postoperative defect following complete Mohs excision of a keratoacanthoma, with an exposed underlying tendon. B. Day 1, following initiation of wound management. C. Day 26, development of granulation tissue with coverage of the previously exposed tendon. D. Day 45, substantial wound contraction with developing epithelial coverage, at which time the patient transitioned to silica-based cream. E. Day 50, complete visible surface coverage. F. Day 118, sustained wound closure on long-term follow-up.

The surgical wound was initially managed with Puracol Plus and petrolatum and was planned for healing by secondary intention. The treating surgeon anticipated that approximately seven weeks would be required for sufficient granulation tissue to cover the exposed tendon. This estimate represented an individualized clinical expectation rather than a prospectively defined endpoint or controlled comparator.

The patient subsequently received an amorphous silica-based fiber wound matrix (SBF; SiOxD Wound Matrix, SiOxMed, Winston-Salem, NC) and SBC (SiOxC Cream, SiOxMed) from SiOxMed for home-based self-treatment [6,7].

During the open-wound phase, the patient rinsed the wound under running tap water once daily and then applied SBF. No secondary gauze, bandage, or occlusive dressing was used. The patient estimated that he used approximately 10 individual 0.3-g units, corresponding to approximately 3 g of SBF in total. After beginning SBF, he discontinued Puracol Plus and petrolatum and did not use topical antibiotics or other wound-care products. He reported no missed applications or treatment interruptions. The patient transitioned from SBF to SBC when the wound surface had become dry, epithelial coverage was evident, and the fibers no longer adhered. The SBC was used during the closed-skin phase and was not applied to the open wound bed.

The patient voluntarily documented the wound course through serial photographs, which were subsequently shared with the authors. These patient-generated images, together with the available clinical information, were retrospectively reviewed for preparation of this case report. The observations were not collected under a prospectively standardized clinical research protocol.

On Day 1, the wound remained open following Mohs excision and was covered with the applied wound material (Figure 1B). By Day 26, the wound bed demonstrated well-developed granulation tissue with coverage of the previously exposed tendons (Figure 1C). This occurred approximately three weeks earlier than the anticipated seven-week period for granulation coverage described to the patient at the outset of secondary-intention management; however, this comparison represents a clinical expectation rather than a controlled comparator.

By Day 45, substantial wound contraction was evident, with a thin epithelial layer developing across the wound (Figure 1D). As the wound surface became dry and the fibers no longer adhered, the patient transitioned from the SBF to SBC for continued home-based skin care.

By Day 50, the wound demonstrated complete visible surface coverage (Figure 1E), and the patient discontinued routine photographic documentation. On Day 118, the patient obtained an additional photograph, which demonstrated sustained closure of the dorsal hand wound (Figure 1F).

During retrospective follow-up, the patient reported no drainage, malodor, erythema, swelling, fever, bleeding, or other suspected complication. He did not require antibiotics, grafting, additional surgery, or unplanned medical care. Wound-related restriction in hand flexion lasted less than one week. He returned to work immediately and reported being able to perform physically demanding activities, including lifting, moving, and packing. Following wound closure, he reported no persistent pain, numbness, restricted motion, contracture, or problematic scarring. These outcomes were retrospectively patient-reported and were not independently assessed using standardized clinical or functional measures.

Written informed consent was obtained from the patient for open-access publication of the de-identified clinical information, patient-generated photographs, retrospective wound analysis, and clinical course described in this report.

## Discussion

This case demonstrates secondary-intention healing of a dorsal-hand defect with an exposed tendon following Mohs surgery and subsequent home-based self-treatment with SBF followed by SBC. Granulation tissue covered the previously exposed tendon by Day 26, substantial wound contraction and epithelial coverage were evident by Day 45, complete visible surface coverage occurred by Day 50, and closure was sustained at Day 118.

Tendon coverage by Day 26 was notable relative to the treating surgeon’s patient-specific expectation that approximately seven weeks would be required for sufficient granulation tissue to cover the exposed tendon, particularly because the patient smoked approximately one pack of cigarettes per day, a recognized risk factor for impaired wound healing. For additional context, Lateo and Langtry prospectively evaluated 28 dorsal-hand surgical defects measuring 15-46 mm in diameter and reported complete healing within 22-63 days, with a median of 44 days among the 24 wounds with available healing data [8]. Complete visible closure by Day 50 in the present case was therefore within the published range. However, Lateo and Langtry reported time to complete healing rather than time to granulation coverage of the exposed tendon, and the treating surgeon’s seven-week estimate was an individualized clinical expectation rather than a controlled comparator. Accordingly, although the observed course was favorable relative to the initial clinical expectation, this case cannot establish that SBF or SBC accelerated healing.

SBF was used during the open-wound phase, whereas SBC was introduced after epithelial coverage developed. Thus, the observed course is most appropriately characterized as granulation, tendon coverage, and contraction during SBF use, followed by SBC use after epithelialization. A conformable fibrous matrix may provide wound coverage and an interface supporting the developing wound bed. Preclinical studies of silica-based fibrous materials have also suggested effects on inflammation, extracellular matrix remodeling, and tissue repair [5]. However, the mechanism and contribution of SBF to healing cannot be determined from this case.

A strength of this report is the use of serial dated photographs documenting the progression of a postoperative dorsal-hand defect with an exposed tendon through granulation coverage, wound contraction, visible epithelial coverage, and sustained closure. Retrospective follow-up also provided relevant information regarding comorbidities, tobacco use, medications, treatment adherence, concomitant wound-care products, suspected complications, functional recovery, and longer-term outcome.

This report also has important limitations. It describes a single uncontrolled case involving retrospective analysis of patient-directed treatment. The patient initially received Puracol Plus and petrolatum before beginning SBF, and the respective contributions of the initial and subsequent treatments cannot be separated. Although the initial defect diameter was documented, serial calibrated measurements of wound area and depth, digital planimetry, validated wound-assessment scores, and standardized functional measurements were not prospectively collected. Treatment adherence, adverse events, and functional outcomes were retrospectively patient-reported and were not independently assessed. The patient smoked approximately one pack of cigarettes per day, representing a potentially important factor affecting wound healing. Patient-generated photographs also varied in positioning, lighting, and scale. Accordingly, neither accelerated granulation nor wound closure can be attributed to SBF or SBC.

## Conclusions

This case demonstrates successful secondary-intention healing of a dorsal hand defect with an exposed tendon following Mohs micrographic surgery and home-based self-treatment with an amorphous SBF wound matrix followed by SBC. Granulation tissue covered the exposed tendon by Day 26, followed by progressive wound contraction and epithelialization, complete visible closure by Day 50, and sustained closure at Day 118. Although causality cannot be established from a single uncontrolled case, the favorable healing trajectory supports prospective evaluation of this treatment approach for postoperative wounds, particularly those involving exposed tendons or other underlying structures.
